# The mutual influences between working memory and empathy for pain: the role of social distance

**DOI:** 10.1093/scan/nsae061

**Published:** 2024-09-06

**Authors:** Ye Yang, Jia Zhao, Huijuan Zhang, Taiyong Bi, Jiangli Tian, Qingqing Li, Cheng Guo

**Affiliations:** Research Center of Mental Health Education, Faculty of Psychology, Southwest University, Chongqing 400715, China; Key Laboratory of Cognition and Personality of the Ministry of Education, Faculty of Psychology, Southwest University, Chongqing 400715, China; CAS Key Laboratory of Mental Health, Institute of Psychology, Chinese Academy of Sciences, Beijing 100101, China; Department of Psychology, University of Chinese Academy of Sciences, Beijing 100101, China; Research Center of Humanities and Medicine, Zunyi Medical University, Zunyi 563003, China; Center for Mental Health Research in School of Management, Zunyi Medical University, Zunyi 563003, China; Center for Mental Health Research in School of Management, Zunyi Medical University, Zunyi 563003, China; Research Center of Mental Health Education, Faculty of Psychology, Southwest University, Chongqing 400715, China

**Keywords:** working memory (WM), empathy for pain (EfP), social distance, event-related potential (ERP), oscillation

## Abstract

Understanding the mechanisms behind the interaction of empathy for pain (EfP) and working memory (WM), particularly how they are influenced by social factors like perceived social distance (SD), is vital for comprehending how humans dynamically adapt to the complexities of social life. However, there is very little known about these mechanisms. Accordingly, we recruited 116 healthy participants to investigate the bidirectional influence and electrophysiological responses between WM and EfP, including the role of SD. Our research results revealed that the interaction between WM load and SD significantly influenced the processing of EfP. Specifically, high WM load and distant SD facilitated early processing of EfP. Conversely, low WM load and close SD promoted late processing of EfP. Furthermore, the interaction between EfP and SD significantly influenced the performance of ongoing WM tasks. Specifically, the kin’s pain, compared to kin’s nonpain, improved the participant’s performance on low-load WM tasks; however, it diminished the participant’s performance on tasks with high WM load. Overall, these results provide evidence at both behavioral and neural levels for the mutual influence of WM and EfP during the same temporal process, and SD emerged as a crucial moderating factor during these mutual influences.

## Introduction

Humans, as social beings, rely on the effective processing of cognitive and affective information within dynamic social environments, which is crucial for adapting to diverse contexts (Tallon 1997, Blair et al. 2007, Liu et al. 2009). Recent research consistently finds that human cognitive and affective processes are influenced by numerous factors (Pessoa 2008, Smith et al. 2014, Hou and Cai 2022). For instance, studies have discovered that working memory (WM) can influence an individual’s empathetic perception of others’ pain (Cui et al. 2017). Conversely, empathy toward others’ pain can impact the processing of WM (Chen et al. 2017). However, these studies often explore the unidirectional relationship between these two processes in isolation. In real life, individuals usually engage in both cognitive and affective processing simultaneously, and as social beings, these processes are also influenced by the interpersonal environments they encounter. Therefore, understanding the psychological and neuro-electrophysiological mechanisms behind the interaction of empathy for pain (EfP) and WM, particularly how they are influenced by social factors like perceived social distance (SD), is vital for comprehending how humans dynamically adapt to the complexities of social life.

When individuals observe others in pain, they vicariously share in the experience and the induced negative emotions, a phenomenon known as EfP (Jackson et al. 2005). WM, as a core cognitive function, refers to the limited resource system in the brain that temporarily stores and processes information during cognitive activities (Meyer et al. 2012). Load theory posits that high cognitive resource engagement reduces the inhibition of irrelevant stimuli (Lavie et al. 2004), meaning that individuals under high WM load are more susceptible to distraction. Studies have revealed that cognitive resource strain affects people’s empathy responses to others’ pain. Specifically, compared to tasks with low WM load, tasks with high WM load enhance emotional arousal and the sharing of others’ pain (Cui et al. 2017). Other studies have discovered that EfP of others affects WM performance. For instance, compared to viewing nonpainful pictures of others, viewing pictures of others who are in pain weakens individuals’ performance in WM tasks (Chen et al. 2017). This effect may be attributed to the activation of common neural representations for self-pain and others’ pain (Meng et al. 2013), as pain, signifying actual or potential injury, disrupts attention. The “threat value of pain” hypothesis suggests that perceiving another’s pain activates survival mechanisms, leading to avoidance behaviors, regardless of objective or subjective danger assessments (Coll et al. 2012). Thus, perceiving another’s pain may induce vicarious anxiety about threats and danger, disrupting attention during WM tasks.

It is noteworthy that, within everyday experiences, the interaction between cognitive and affective processes in individuals deserves closer scrutiny, as these processes might inter-relate rather than function separately. Some studies have examined the effects of WM on EfP and the impact of EfP on WM in separate experimental settings (Chen et al. 2017, Cui et al. 2017). However, the bidirectional influence of WM on EfP response and EfP on WM performance within the same timeframe remains unexplored. For instance, does a doctor’s empathic response to seeing a stranger patient injured and bleeding differ when the doctor is under high work pressure compared to when they are relaxed and free of tasks? Additionally, if the doctor witnesses the injury and bleeding and then continues with a high cognitive load task, will their efficiency and performance be affected? Furthermore, if the injured person is the doctor’s relative, will the empathic response and subsequent cognitive performance differ from those elicited by a stranger?

Humans, being inherently social, often find themselves in environments replete with diverse interpersonal relationships. An individual’s cognitive and affective processes are influenced, to some extent, by the perceived SDs within these contexts (Nissan et al. 2015). SD encompasses an individual’s perception or experience of distance from others (Li et al. 2020a), with varying levels among kin and strangers. Research indicates that participants rate higher pain intensity when imagining a loved one in pain compared to a stranger, with stronger brain region activations related to EfP (Cheng et al. 2010). Similar studies have shown stronger left anterior insula activation when observing pain in the same social group versus different groups (Sheng and Han 2012). Both pain and EfP are associated with this brain region (Decety et al. 2012). Recent studies also demonstrate that empathy is foundational for altruistic behavior, biased toward socially close individuals (Fowler et al. 2021). Kin selection theory suggests that through empathy for kin, we can better meet their needs and engage in altruistic behavior, thereby increasing their chances of survival. This contributes to the survival and reproduction of kin, indirectly promoting the transmission of our own genes (Hamilton 1964). Thus, empathic responses to kin are typically stronger than those to strangers. In parallel, more distant SD accompanied by less worry and anxiety would then result in more abstract thinking (Smith and Trope 2006, Trope and Liberman 2010), which, in turn, facilitates the organization and manipulation of complex information, improving WM capabilities (Herzmann et al. 2011).

Based on the preceding research, it is clear that exploring the mutual influence of WM and EfP, as well as the interplay further resulting from the impact of SD, aids in our enhanced understanding of how individuals concurrently engage in cognitive and affective processing to adapt to complex social environments in daily life. Therefore, in this study, we employed electroencephalography (EEG)/event-related potential (ERP) to investigate how WM and EfP are mutually influential, while exploring the role of SD, which has practical significance. Since EEG/ERP measurement offers a window into the temporal dynamics of neural responses to observing others’ pain and memory judgment, we gathered participants and compared their behavioral and EEG/ERP responses while they completed tasks that combined EfP and WM, where they processed pain perception and made memory judgments. According to earlier ERP research on EfP (Fan and Han 2008, Decety et al. 2010, Meng et al. 2013), sharing others’ emotions regarding EfP is linked to the early impacts of pain in the fronto-central N2 component. Moreover, it is generally accepted that the later effects of pain in the centro-parietal P3 and late positive potential (LPP) components represent prolonged attentional processing and cognitive appraisal of stimuli with motivational importance. Pain impacts on these ERP components have been linked to processes of others’ pain that are early automatic and late controlled (Fan and Han 2008, Cheng et al. 2014, Fabi and Leuthold 2017). Furthermore, after being exposed to others’ painful situations as opposed to nonpainful situations, there have been increased theta event-related synchronization (θ-ERS) responses seen over parietal areas (Mu et al. 2008). In the context of WM tasks, previous research has suggested that a larger P2 component may reflect increased attentional demands for target stimuli (Gevins et al. 1996, Lijffijt et al. 2009, Lubitz et al. 2017). Also, memory studies on the N3 component have shown it to be a measure of “object recognition,” where harder target identifications result in larger N3 amplitudes (Maguire et al. 2013). On the other hand, in memory judgment tasks, greater alpha event-related desynchronization (α-ERD) is associated with better performance because it links enhanced cognitive processing in the brain regions relevant to the task (Pesonen et al. 2007, Deiber et al. 2015, Scharinger et al. 2017).

According to existing research findings, we propose the following hypotheses: during the EfP phase: (I) drawing from kin selection theory (Hamilton 1964), we hypothesize that the intensity and unpleasantness of EfP for the kin’s pain will be significantly higher than those for a stranger’s pain, regardless of whether the WM load is high or low. Additionally, the kin’s pain will elicit larger N2, P3, and LPP amplitudes, as well as greater θ-ERS magnitudes. (II) According to load theory (Lavie et al. 2004), we hypothesize that under high WM load, EfP responses to a stranger’s pain will be stronger compared to low WM load, with larger N2, P3, and LPP amplitudes. In contrast, due to the influence of kinship, EfP responses and electrophysiological reactions to the kin’s pain will not be affected by the levels of WM load. During the memory judgment phase, (III) based on the “threat value of pain” hypothesis (Coll et al. 2012), we hypothesize that, compared to EfP responses to a stranger’s pain, EfP responses to a kin’s pain will result in lower accuracy (ACC) and longer reaction times (RTs) in high-load WM letter judgments, with larger P2 and N3 amplitudes and lower α-ERD magnitudes. Low-load WM letter judgments will not be affected by SD. (IV) We hypothesize that the kin’s pain, compared to the absence of the kin’s pain, is expected to lead to lower ACC and longer RTs in high-load WM letter judgments, with larger P2 and N3 amplitudes.

## Materials and methods

### Participants

An *a priori* power analysis was conducted using G*Power 3.1.9.7. The selected analysis type was a repeated measures analysis of variances (ANOVA) with three within-subject factors: WM load (low and high), SD priming (stranger and kin photos), and picture painfulness (painful and nonpainful). Based on the experimental design and prior research, the power analysis indicated that a minimum of 16 participants would be required to achieve a power of 0.8, an alpha of 0.05, and a medium effect size (Faul et al. 2007, Ren et al. 2022). The G*Power output is included in the Supplementary materials under the title “GPower_Output.” Furthermore, recent evidence has shown that neuroscience investigations are typically underpowered, leading to a dual issue of overestimation of effects that are observed and a lower likelihood of finding an effect (Boudewyn et al. 2018, Coll 2018). Thus, to ensure the stability of the results, 116 participants (72 females; mean age: 20.63 ± 1.59 years; range: 18–28 years) took part in the study for payment (50 RMB/h) or student credits (two extracurricular activity credits per hour). Each participant was right-handed, with normal or corrected-to-normal vision, without color blindness, had current or past psychiatric or neurological disorders, and was not using any medication. The Southwest University Ethics Committee approved the experimental protocols, and each participant completed an informed consent form.

### Questionnaires

Participants completed the Chinese version of the Interpersonal Reactivity Index (IRI) prior to the experiment to measure their levels of empathy (Davis 1983, Zhang et al. 2010). This scale demonstrated adequate internal consistency (ranging from 0.53 to 0.78) and test–retest reliability (ranging from 0.56 to 0.82) (Song et al. 2016). This IRI consists of four subscales: perspective-taking (PT), fantasy (FS), empathic concern (EC), and personal distress (PD). PT measures an individual’s ability to understand the thoughts and viewpoints of others in everyday life. FS assesses the capacity of a person to place themselves in the situation of fictional characters and experience their emotions and thoughts. Higher scores in PT and FS indicate better cognitive empathy abilities. EC measures a person’s sympathy and concern for others when they encounter difficulties, reflecting an emotional resonance and care for the misfortune of others. PD evaluates the discomfort and stress an individual feels upon witnessing others in distress or experiencing pain. It also involves how individuals handle and respond to the personal negative emotions that arise from emotional resonance. Higher scores in EC and PD indicate better affective empathy capabilities.

The Inclusion of Other in the Self (IOS) Scale was used to measure the SD between the self and the kin to make sure that the kin is the participant’s favorite and closest one (Aron et al. 1992, Tan et al. 2015). The IOS Scale showed strong validity (as expected, it was favorably associated with the Subjective Closeness Index and the Sternberg Intimacy Scale) and reliability (*α* = 0.95; test–retest reliability was 0.85) (Aron et al. 1992). The scale consists of seven pairs of circles, each representing a target person and the self. The degree of intersection between the circles represents the level of SD (range from 1 to 7; 1 = extremely far away, 7 = very close). The participants were told to pick a pair of circles that best represented their interactions with each target individual. The mean score obtained through the IOS measurement for all participants’ closest one was 5.53 ± 0.92. According to previous research, a high IOS score denotes a socially close distance between the participant and the target person (Aron et al. 1992, Wu et al. 2011). Using the photos of 40 stranger’s faces in our experiment, prior to the commencement of the study, participants were instructed to verify that they had never encountered or were acquainted with the subjects of the photos.

### Stimulus and task

We combined a classical task of EfP with a WM task (MacNamara et al. 2012, Wang et al. 2016, Cui et al. 2017) to explore the mutual influence of EfP and WM, as well as the role played by SD.

#### Visual stimuli

##### Facial photos

We captured neutral faces of strangers using a camera and used Photoshop to remove parts below the neck, resulting in 40 color photos (20 females) that were consistent in physical properties such as size (12.66 × 11.64 cm), resolution (359 × 330 pixels), brightness, and contrast. Before the experiment, we asked participants to provide a photo of a neutral face of their favorite and closest kin; these photos of kin were then modified using Photoshop to ensure that the physical attributes such as the size, brightness, and contrast of the photos were consistent with those of the strangers’ faces.

##### Painful and nonpainful pictures.

In our study, we primarily used 80 pictures: 40 depicting painful situations and 40 depicting nonpainful situations involving limbs (Meng et al. 2024). Each picture featured a body part (e.g. hand, forearm, or foot) in a commonplace situation that was either experiencing pain (e.g. a hand injury resulting from improper fruit cutting with a knife) or not experiencing pain (e.g. proper fruit cutting with a knife, resulting in no injury). These pictures were previously published in an ERP investigation of empathic responses to human suffering (Meng et al. 2013). The pairs of painful and nonpainful pictures were meticulously matched for luminance, contrast, and color, and all pictures were standardized to 9 × 6.76 cm (width × height) with a resolution of 394 × 296 pixels.

#### Study design

A within-subject design with the following factors was used: 2 (WM load: low and high loads) × 2 (SD priming: stranger and kin photos) × 2 (picture painfulness: painful and nonpainful pictures). WM load, the first experimental factor, had two levels: high and low. The length of a string of letters needed to be memorized served to operationalize WM load levels. The letter length for the high-load WM level was six, while that of the low-load WM level was two (Wang et al. 2016, Cui et al. 2017). The second experimental factor, SD priming, comprised two levels: close distance (the photo of kin) and distant distance (the photo of a stranger). The third experimental factor, the stimuli inducing empathy, also included two levels: painful pictures and nonpainful pictures. The experiment consisted of four blocks, each comprising 80 trials. The eight conditions were presented pseudo-randomly and with equal frequency, resulting in a total of 320 trials. During the experiment, each face photo of a stranger and an empathy picture appeared four times, while the pictures of a close kin appeared 160 times in total to maintain uniformity among the conditions.

#### Experimental procedures

Before the formal experiment, 10 trials were conducted on each of the participants. During this process, participants had to learn the procedural details of the experiment and achieve a proficient level before moving on to the formal experiment. Once the experimenter confirmed that the participant had learned the procedure, the experiment entered the formal phase. The total duration of the practice session combined with the tasks in the formal experiment is approximately 80 min. Taking into account the breaks for the participants, the entire experiment takes about 100 min to complete.

**Figure 1.**
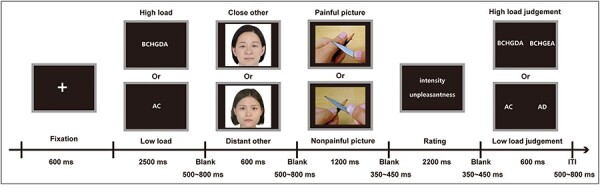
Experimental design.

As illustrated in Fig. 1, each trial began with a white fixation cross presented in the center of the computer screen for 600 ms at a viewing distance of about 80 cm, followed by the presentation of a string of letters that needed to be memorized by the participant lasting 2500 ms. Subsequently, a blank screen was presented for 500–800 ms, after which a facial photo was displayed for 600 ms. Following the disappearance of the facial photo, another blank screen was presented for 500–800 ms and then an empathy-inducing picture was displayed for 1200 ms. This was followed by a sequence of a blank screen for 350–450 ms and a rating screen for 2200 ms, during which the participant was required to verbally rate the intensity and unpleasantness based on the following two questions (during the experiment, these two questions were presented in Chinese): (I) “Just watch carefully and imagine: if you are the person in the face photo, how intense is the pain you feel when you experience the scene presented in this picture?” 0–10: no sensation—the strongest sensation imaginable and (II) “Just watch carefully and imagine: if you are the person in the face photo, how unpleasant would you feel if you experienced the scene presented in the picture?” 0–10: not unpleasant—extremely unpleasant. Intensity and unpleasantness ratings focused on the participants’ perceptions of sensory-discriminative and affective-motivational aspects, respectively (Kim et al. 2017). Following the disappearance of the rating interface, two strings of letters of identical length were presented simultaneously on the left and right sides of the screen, with one string being identical to the target string that was previously memorized by the participant. The participants were then told to choose a key in this interface—press the “F” key if they thought the letter strings on the left matched what they had previously memorized or press the “J” key if they thought the letter strings on the right matched what they had previously memorized. At the same time, the RTs of key and ACC of the participants in the judgment stage were recorded using E-Prime 3.0 software. The judgment screen vanished after the key was pressed, and the next trial started.

Each trial commenced with the presentation of a fixation cross lasting for 600 ms, followed by target letter strings of either six or two letters, displayed for 2500 ms. Participants were required to memorize these target letter strings. Following this, a blank screen appeared for 500–800 ms, during which a facial photo (stranger or kin) was presented for 600 ms before the subsequent blank screen vanished. Afterward, empathy photos, categorized as either painful or nonpainful, were presented for 1200 ms, followed by another blank display. Next, participants were told to orally score the perceived intensity and unpleasantness induced by the empathy photos using 11-point numerical rating scales, with a rating period lasting 2200 ms. Following the rating process, a blank screen appeared, leading to the subsequent memory judgment phase. During this phase, a series of letters simultaneously appeared on both sides of the computer screen. Participants were required to determine which side’s letter strings matched the target letter strings they had memorized. The keyboard response disappeared at this point. The intertrial interval (ITI) lasted between 500 and 800 ms.

### EEG recording

EEG data between direct current and 100 Hz was continuously recorded using the Brain Product System (Brain Products GmbH) at a sampling rate of 500 Hz. The standard 10–20 system was used to place the 64 Ag–AgCl scalp electrodes. Online reference was made using the FCz electrode. All impedances were kept below 10 kΩ. Using an IO electrode positioned on the left lower eyelid, an electro-ocular signal was simultaneously captured to track eye movements and blinking.

### Time-domain data analysis

MATLAB R2022b (MathWorks, USA) and the EEGLAB toolbox v2022.1 were used to preprocess and analyze the acquired EEG data (Delorme and Makeig 2004). As we were concerned with the interactive effects of WM load and SD on EfP response, as well as the impact of EfP and SD on the performance of WM, we restricted our analysis to the EEG signals during the presentation of the empathy pictures and memory judgment phases. To extract relevant information, the continuous EEG signals were band-pass filtered (1–40 Hz) and segmented into 1200-ms time windows. Following this, time windows of 200 ms before and 1000 ms after the onset of stimuli were extracted and baseline corrected using the 200-ms interval before the stimulus onset. Additionally, a visual inspection was conducted to eliminate trials that contained significant noise resulting from gross movements (Ren et al. 2022, Zhang et al. 2022). The proportion of excluded trials was restricted to not more than 1% of the total trials. Consequently, not more than four trials were excluded for each condition, ensuring that a minimum of 36 trials per condition remained for the final analysis. The independent component analysis algorithm was used to correct artifacts associated with ocular movements and eye blinks (Jung et al. 1998).

According to earlier EfP investigations (Decety et al. 2010; Li et al. 2020b, Meng et al. 2020), scalp topographies of grand average ERP activity and dominant ERP components, including N2 and LPP, were detected during the presentation of empathy pictures. The average peak amplitude of N2 was assessed within 200–440 ms after the start of the empathy stimulation at frontal electrodes (F3, F4, Fz, F1, and F2). The average peak amplitude of P3 and the mean LPP amplitude were recorded 280–420 ms and 450–700 ms, respectively, after the start of the visual stimulation at the parietal–central electrodes (P1, Pz, and P2). Prominent ERP components during memory judgment, specifically N3 and P2, were identified based on the scalp topographies of grand average ERP activity and previous WM studies (Yuan et al. 2016, Getzmann et al. 2018). Accordingly, the average peak amplitudes of the N3 components were measured at F1, Fz, and F2 within the time windows of 180–380 ms. We also recorded the average peak amplitude of the P2 component from 160 to 250 ms at three specific electrodes, namely, C3, Cz, and C4.

### Time-frequency analysis

The EEG time courses elicited by the presentation of empathy stimuli and memory judgment letter strings were transformed into the time-frequency domain to identify EEG oscillatory responses associated with EfP response and the performance of WM. To get time-frequency distributions (TFDs) of the EEG time courses, a windowed Fourier transform with a fixed 200-ms Hanning window was specifically applied (Zhang et al. 2012). With time ranging from −200 to 1000 ms (in 2-ms intervals) and frequency ranging from 1 to 30 Hz (in 1-Hz intervals), each time course was subjected to a windowed Fourier transform. This produced a complicated time-frequency estimate at each point on the time-frequency plane. At each time-frequency point, the resulting spectrogram depicted signal power as a combined function of time and frequency. The spectrograms were baseline corrected using a subtraction approach [*P*(*t*_1_, *f*) − mean (*R*(*t*_2_, *f*))] with a reference interval of −200 to 0 ms relative to the commencement of empathy or memory judgment stimulation.

Previous research has demonstrated that greater θ-ERS response occurs over the parietal regions after exposure to others’ painful situations than after nonpainful situations (Mu et al. 2008) and that greater α-ERD response is more pronounced in parietal–central regions during memory judgment tasks (Pesonen et al. 2007, Deiber et al. 2015, Scharinger et al. 2017). Thus, during the empathy phase for each participant and stimulation condition, we measured θ-ERS magnitudes at parietal electrodes (P1, Pz, and P2) by averaging the oscillation magnitudes in the 3–5 Hz frequency range and within 100–350 ms after the empathy stimuli. During memory judgment tasks, we measured α-ERD magnitudes at parietal–central electrodes (CP3, CP1, CPz, CP2, CP4, P3, P1, Pz, P2, and P4) by averaging the oscillation magnitudes in the 9–15 Hz frequency range and within 300–800 ms after the memory judgment stimulation onset. In these two stages, the frequency and time range for the time-frequency analysis are primarily determined using a data-driven approach, focusing on selecting regions that exhibit the most significant differences from the baseline for further analysis and statistical examination. Scalp topographies of θ-ERS and α-ERD magnitudes were computed by spline interpolation.

### Statistical analysis

To conduct the statistical analysis, SPSS 26.0 (IBM Corp., New York, NY) was used. Three-way repeated measures ANOVA with three within-subject factors, WM load (low vs. high), SD priming (stranger vs. kin), and picture painfulness (painful vs. nonpainful), were used on the subjective ratings, i.e. pain intensity and unpleasantness; the time-domain responses, i.e. peak amplitudes of N2 and P3 and mean amplitudes of LPP components; and the time-frequency responses, i.e. the magnitudes of θ-ERS, in the empathic section to compare the effects of interactions between WM load and SD regarding the response of different picture painfulness. For the WM behavioral data (RT and ACC), time-domain responses, i.e. peak amplitudes of N3 and P2 components; time-frequency responses, i.e. the magnitudes of α-ERD, during the memory judgment phase; and three-way repeated measures ANOVA with three within-subject factors, WM load (low vs. high), SD priming (stranger vs. kin), and picture painfulness, (painful vs. nonpainful), were also performed to assess the effects of interaction between picture painfulness and SD priming on performances of different WM loads. If any main effect or interaction effect was discovered, *post hoc* comparisons were conducted and Bonferroni corrections were used for multiple comparisons (Ren et al. 2022). To examine the correlation between empathy traits and behavioral and neural responses to EfP, we employed Spearman correlation analyses in SPSS.

## Results

### EfP phase

#### Behavioral results

Supplementary Table S1 (Supplementary Materials) provides descriptive statistics (*M* ± SE) of the average intensity and unpleasantness in response to painful and nonpainful pictures in all situations. Supplementary Table S2 (Supplementary Materials) contains a complete list of all statistical comparisons.

For pain intensity, a three-way repeated measures ANOVA revealed significant main effects of distance [*F*(1115) = 32.41, *P* < .001, *η*_p_^2^ = 0.220] and painfulness: [*F*(1115) = 1789.76, *P* < .001, *η*_p_^2^ = 0.940]. There were significant interactions between distance and painfulness: [*F*(1115) = 25.21, *P* < .001, *η*_p_^2^ = 0.180]. A subsequent analysis showed that after kin priming, participants rated higher pain intensity for both painful pictures (kin priming: 6.115 ± 0.090; stranger priming: 5.950 ± 0.088; *P* < .001) and nonpainful pictures (kin priming: 1.824 ± 0.058; stranger priming: 1.783 ± 0.058; *P* = .003) than after stranger priming.

For the unpleasantness rating, a three-way repeated measures ANOVA revealed significant main effects of distance [*F*(1115) = 14.20, *P* < .001, *η*_p_^2^ = 0.110] and painfulness [*F*(1115) = 1299.42, *P* < .001, *η*_p_^2^ = 0.919]. There were significant interactions between load and distance [*F*(1115) = 4.21, *P* = .042, *η*_p_^2^ = 0.035] and between distance and painfulness [*F*(1115) =14.26, *P* < .001, *η*_p_^2^ = 0.110] as well. A subsequent analysis showed that after kin priming, participants reported higher unpleasantness ratings under both high-load situations (kin priming: 3.265 ± 0.096; stranger priming: 3.199 ± 0.093; *P* = .028) and low-load situations (kin priming: 3.292 ± 0.092; stranger priming: 3.169 ± 0.090; *P* < .001) than after stranger priming. Participants also reported higher unpleasantness ratings after kin priming for painful pictures (kin’s pictures: 5.701 ± 0.144; stranger’s pictures: 5.532 ± 0.144; *P* < .001) than after stranger priming.

#### EEG time-domain results

**Figure 2.**
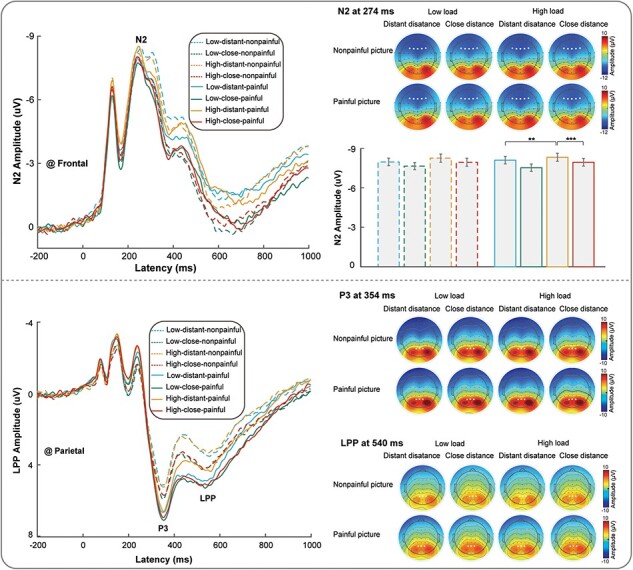
ERP responses during the EfP phase.

Supplementary Table S1 provides descriptive statistics (*M* ± SE) of the average peak amplitudes of N2, P3, and mean amplitudes of LPP, in response to painful and nonpainful pictures in all situations. Supplementary Table S2 contains a complete list of all statistical comparisons. Grand average time-domain activity during the empathy conditions is shown in Fig. 2.

ERP waveforms and scalp topographies were recorded to investigate the participant’s responses to painful (solid lines) or nonpainful (dashed lines) pictures under various experimental conditions, including low-load distant distance priming, low-load close distance priming, high-load distant distance priming, and high-load close distance priming. The waveforms were measured at frontal electrodes (F3, F1, Fz, F2, and F4) and parietal electrodes (P1, Pz, and P2). The peak amplitudes of dominant components, namely, N2, P3, and mean amplitudes of LPP, evoked by painful and nonpainful pictures, were compared across the different experimental conditions. The electrodes used to evaluate the ERP amplitudes were marked with enlarged dots on the corresponding scalp topographies. The data are presented as *M* ± SE. Statistical significance was denoted as **P < .01, and ***P < .001.

For N2 amplitudes, three-way repeated measures ANOVAs revealed significant main effects of distance [*F*(1115) = 27.33, *P* < .001, *η*_p_^2^ = 0.192]. There was significant three-way interaction between load, distance, and painfulness [*F*(1115) = 8.92, *P* = .003, *η*_p_^2^ = 0.072]. We conducted three two-way repeated measures ANOVAs with two factors by fixing another factor to understand the three-way interaction. We discovered three significant two-way interactions: (I) for both painful and nonpainful pictures, there were two significant interactions between load and distance [*F*(1115) = 4.41, *P* = .038, *η*_p_^2^ = 0.037] and *F*(1115) = 4.32, *P* = .040, *η*_p_^2^ = 0.036, respectively]; (II) for low load, there was a significant interaction between distance and painfulness [*F*(1115) = 10.09, *P* = .002, *η*_p_^2^ = 0.081]; and (III) for stranger priming, there was a significant interaction between load and painfulness [*F*(1115) = 8.73, *P* = .004, *η*_p_^2^ = 0.071]. We were particularly interested in the interaction between load and distance among these significant two-way interactions. As a result, we analyzed the interaction between load and distance further. The results revealed that, for painful pictures, participants had a larger N2 amplitude under high-load situations than low-load situations after the stranger priming (high load: −10.876 ± 0.436; low load: −10.386 ± 0.414; *P* = .004), whereas no such significant difference in N2 amplitude was seen for the kin priming (high load: −10.253 ± 0.416; low load: −10.173 ± 0.423; *P* = .658). In addition, for painful pictures, participants had a larger N2 amplitude under high-load situations after the stranger priming than after the kin priming (stranger priming: −10.876 ± 0.436; kin priming: −10.253 ± 0.416; *P* < .001), whereas no such significant difference in N2 amplitude was seen for the low load (stranger priming: −10.386 ± 0.414; kin priming: −10.173 ± 0.423; *P* = .198). For P3 amplitudes, three-way repeated measures ANOVA revealed significant main effects of distance [*F*(1115) = 19.77, *P* < .001, *η*_p_^2^ = 0.147] and painfulness [*F*(1115) = 147.40, *P* < .001, *η*_p_^2^ = 0.562], respectively, but no interaction effects. This indicated that under both the kin’s photo priming, painful pictures elicited larger P3 amplitudes, respectively. For LPP mean amplitudes, a three-way repeated measures ANOVA revealed significant main effects of load [*F*(1115) = 4.00, *P* = .048, *η*_p_^2^ = 0.034], distance [*F*(1115) = 72.80, *P* < .001, *η*_p_^2^ = 0.388], and painfulness [*F*(1115) = 103.43, *P* < .001, *η*_p_^2^ = 0.474], but no significant interaction effects. This indicates that under a low load, kin priming and painful pictures elicited larger LPP mean amplitudes.

#### EEG time-frequency results

**Figure 3.**
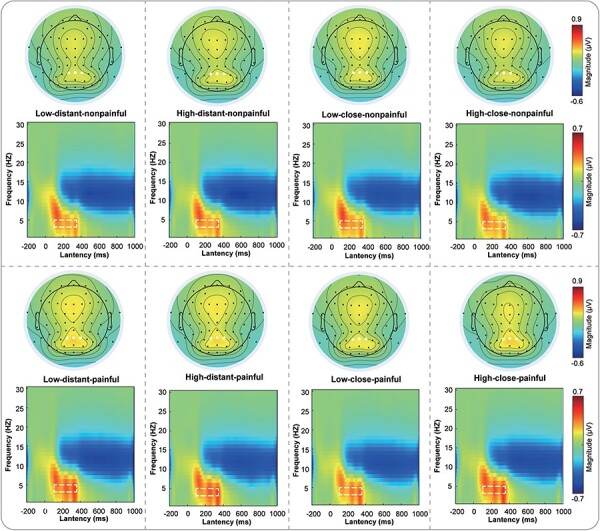
Time-frequency responses during the EfP phase.

Supplementary Table S1 provides descriptive statistics (*M* ± SE) of the θ-ERS magnitudes in response to painful and nonpainful pictures in all situations. Supplementary Table S2 contains a complete list of all statistical comparisons. Grand average TFDs during the EfP phase are shown in Fig. 3.

TFDs of neural responses were examined for low-load distant distance, low-load close distance, high-load distant distance, and high-load close distance priming conditions in response to nonpainful and painful pictures during the EfP phase. The color scale represents the relative increase or decrease in oscillatory magnitude compared to a prestimulus interval (−200 to 0 ms). The signals were measured at parietal electrodes (P1, Pz, and P2). Both nonpainful and painful pictures elicited a sustained θ-ERS response, characterized by oscillations in the theta frequency range (3–5 Hz) and a latency window of 100–350 ms, indicated by dashed line rectangles. The electrodes used to evaluate the θ-ERS magnitude were marked with enlarged dots on the scalp topographies.

For θ-ERS, a three-way repeated measures ANOVA revealed significant main effects of painfulness [*F*(1115) = 25.29, *P* < .001, *η*_p_^2^ = 0.180]. There were significant three-way interactions among load, distance, and painfulness [*F*(1115) = 5.40, *P* = .022, *η*_p_^2^ = 0.045] as well. We conducted three two-way repeated measures ANOVAs with two factors by fixing another factor to understand the three-way interaction. We discovered significant two-way interactions: for kin priming, there was a significant interaction between load and painfulness [*F*(1115) = 6.19, *P* = .014, *η*_p_^2^ = 0.051]. In a more detailed analysis of the interaction effects, we observed that during kin priming, participants exhibited significantly greater θ-ERS magnitudes when exposed to painful pictures under conditions of high cognitive load, as compared to conditions of low cognitive load (high load: 0.358 ± 0.025; low load: 0.311 ± 0.027; *P* = .033), whereas no such significant difference in θ-ERS magnitudes was seen for viewing nonpainful pictures (high load: 0.279 ± 0.022; low load: 0.303 ± 0.023; *P* = .163).

#### Correlation analyses

**Figure 4.**
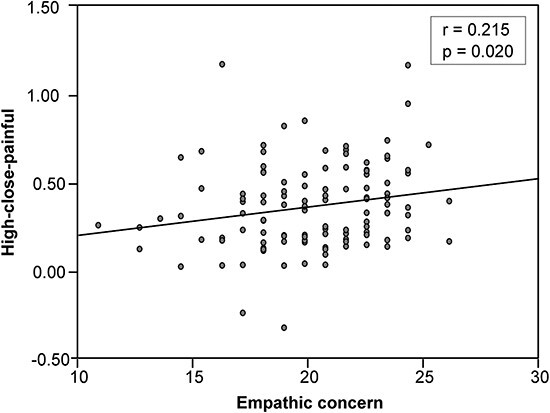
Spearman correlation between θ-ERS and EC.

We conducted Spearman correlation analyses to examine the relationship between all values of dependent variable in response to empathy pictures and four subscale scores of the IRI questionnaire. The results revealed a significant positive correlation (*r* = 0.215, ***P*** = .020) between the θ-ERS induced by processing painful pictures under high load after kin priming and the EC subscale of the IRI (Fig. 4).

θ-ERS induced by processing painful pictures under high cognitive load after kin priming during the EfP phase is shown in relation to the EC subscale of the IRI.

### Memory judgment phase

#### Behavioral results

Supplementary Table S3 (Supplementary Materials) provides descriptive statistics (*M* ± SE) of the mean RT and ACC in response to low and high loads during the memory judgment phase. Supplementary Table S4 (Supplementary Materials) contains a complete list of all statistical comparisons.

For RT, a three-way repeated measures ANOVA revealed significant main effects of load [*F*(1115) = 205.23, *P* < .001, *η*_p_^2^ = 0.641] and painfulness [*F*(1115) = 36.16, *P* < .001, *η*_p_^2^ = 0.239]. There were also significant interactions between load and distance [*F*(1115) = 17.50, *P* < .001, *η*_p_^2^ = 0.132] and between distance and painfulness [*F*(1115) = 6.68, *P* = .011, *η*_p_^2^ = 0.055]. We were particularly interested in the interaction between distance and painfulness between these significant two-way interactions. As a result, we performed the subsequent analysis which discovered that participants had shorter RT after viewing nonpainful pictures for memory judgment stimuli than after viewing a painful picture under both kin priming (painful pictures: 1686.040 ± 48.020; nonpainful pictures: 1623.450 ± 44.310; *P* = .001) and stranger priming (painful pictures: 1727.770 ± 49.770; nonpainful pictures: 1605.212 ± 45.950; *P* < .001). At the same time, for painful pictures, participants had shorter RT during kin priming for memory judgment stimuli (kin’s pictures: 1686.040 ± 48.020; stranger’s pictures: 1727.770 ± 49.770; *P* = .011) than during stranger priming.

**Figure 5.**
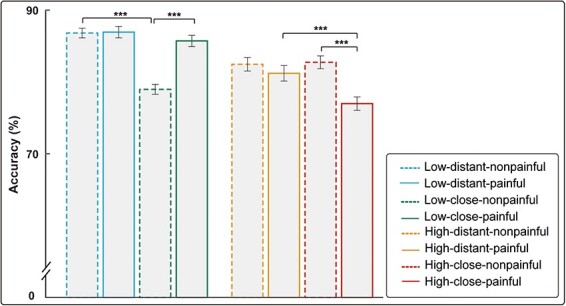
ACC for letter strings during the memory judgment phase.

For ACC, a three-way repeated measures ANOVA revealed significant main effects of load [*F*(1115) = 40.44, *P* < .001, *η*_p_^2^ = 0.260] and distance [*F*(1115) = 85.40, *P* < .001, *η*_p_^2^ = 0.426]. There were also significant interactions between load and distance [*F*(1115) = 13.16, *P* < .001, *η*_p_^2^ = 0.103] and between load and painfulness [*F*(1115) = 91.60, *P* < .001, *η*_p_^2^ = 0.443]. There was a significant three-way interaction among load, distance, and painfulness [*F*(1115) = 81.76, *P* < .001, *η*_p_^2^ = 0.416] for memory judgment stimuli. We conducted three two-way repeated measures ANOVAs with two factors by fixing another factor to understand the three-way interaction. We discovered three significant two-way interactions: (I) for both painful and nonpainful stimuli, there were two interactions between load and distance [*F*(1115) = 8.76, *P* = .004, *η*_p_^2^ = 0.071) and *F*(1115) = 90.22, *P* < .001, *η*_p_^2^ = 0.440, respectively]; (2) for both high load and low load, there were two significant interactions between distance and painfulness [*F*(1115) = 16.87, *P* < .001, *η*_p_^2^ = 0.128) and *F*(1115) = 54.95, *P* < .001, *η*_p_^2^ = 0.323, respectively]; and (3) for kin priming, there was a significant interaction between load and painfulness [*F*(1115) = 187.78, *P* < .001, *η*_p_^2^ = 0.620]. We were particularly interested in the interaction between distance and painfulness among these significant two-way interactions. Accordingly, we analyzed the interaction between distance and painfulness. The results revealed that for low load, under kin priming, participants had higher ACC after viewing the painful pictures than after viewing the nonpainful ones (painful pictures: 85.7% ± 0.8%; nonpainful pictures: 79.0% ± 0.7%; *P* < .001), whereas no such significant difference was seen under stranger priming (painful pictures: 86.9% ± 0.8%; nonpainful pictures: 86.8% ± 0.7%; *P* = .840). For low load, under kin priming, participants had lower ACC than stranger priming after viewing the nonpainful pictures (kin priming: 79.0% ± 0.7%; stranger priming: 86.8% ± 0.7%; *P* < .001), whereas no such significant difference was seen after the painful pictures (kin priming: 85.7% ± 0.8%; stranger priming: 86.9% ± 0.8%; *P* = .077). For high load, under kin priming, participants had a lower ACC after viewing the painful pictures than after viewing the nonpainful pictures (painful pictures: 77.0% ± 0.9%; nonpainful pictures: 82.7% ± 0.9%; *P* < .001), whereas no such significant difference was seen for stranger priming (painful pictures: 81.2% ±1.1%; nonpainful pictures: 82.5% ± 0.9%; *P* = .117). At the same time, for high load, under the kin priming, participants had lower ACC after viewing the painful pictures (kin priming: 77.0% ± 0.9%; stranger priming: 81.2% ± 1.1%; *P* < .001) than under stranger priming, whereas no such significant difference was seen for the nonpainful pictures (kin priming: 82.7% ± 0.9%; stranger priming: 82.5% ± 0.9%; *P* = .695). A comparison of the ACC among different conditions during the memory judgment phase is shown in Fig. 5.

ACC, one of the behavioral responses, was examined for distant distance nonpainful picture, distant distance painful picture, close distance nonpainful picture, and close distance painful picture conditions in response to low-load letter strings and high-load letter strings during the memory judgment phase. Statistical significance was denoted as *P < .05, **P < .01, and ***P < .001.

#### EEG time-domain results

**Figure 6.**
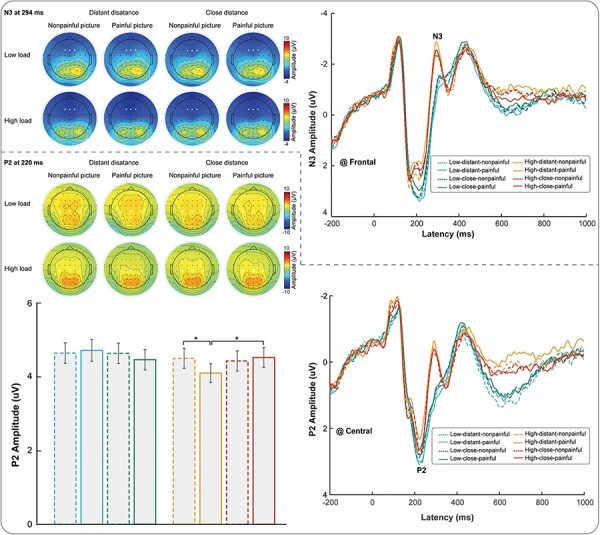
ERP responses during the memory judgment phase.

Supplementary Table S3 provides descriptive statistics (*M* ± SE) of the average peak amplitudes of N3 and P2 in response to memory judgment stimuli under all conditions. Supplementary Table S4 contains a complete list of all statistical comparisons. Grand average ERP activity during the memory judgment phase is shown in Fig. 6.

ERP waveforms and scalp topographies were recorded for distant distance nonpainful picture, distant distance painful picture, close distance nonpainful picture, and close distance painful picture conditions in response to low-load letter strings and high-load letter strings during the memory judgment phase. The waveforms were measured at frontal electrodes (F1, Fz, and F2) and central electrodes (C3, Cz, and C4). The peak amplitudes of dominant components, namely, N3 and P2, evoked by low- and high-load letter strings, were compared across the different experimental conditions. The electrodes used to evaluate the ERP amplitudes were marked with enlarged white dots on the corresponding scalp topographies. The data are presented as *M* ± SE. Statistical significance was denoted as *P < .05, **P < .01, and ***P < .001.

For N3 amplitudes, a three-way repeated measures ANOVA revealed a significant main effect of load [*F*(1115) = 9.24, *P* = .003, *η*_p_^2^ = 0.074]. This indicated that high-load conditions elicited larger N3 amplitudes. For P2 amplitudes, a three-way repeated measures ANOVA revealed no significant main effect. However, there were significant three-way interactions among load, distance, and painfulness [*F*(1115) = 5.30, *P* = .023, *η*_p_^2^ = 0.044]. We conducted three two-way repeated measures ANOVAs with two factors by fixing another factor to understand the three-way interaction which revealed two significant two-way interactions: (I) for painful pictures, there was a significant interaction between load and distance [*F*(1115) = 8.51, *P* = .004, *η*_p_^2^ = 0.069] and (II) for high load, there was a significant interaction between distance and painfulness [*F*(1115) = 4.31, *P* = .040, *η*_p_^2^ = 0.036]. We were particularly interested in the interaction between distance and painfulness between these significant two-way interactions. As a result, we analyzed the interaction between distance and painfulness further. The results revealed that for high load, under the stranger priming, participants had a larger P2 amplitude after viewing the nonpainful pictures than after viewing the painful pictures (painful pictures: 4.076 ± 0.254; nonpainful pictures: 4.473 ± 0.269; *P* = .019), whereas no such significant difference in P2 amplitude was seen for the kin priming (painful pictures: 4.497 ± 0.266; nonpainful pictures: 4.404 ± 0.273; *P* = .572). At the same time, for high load, under kin priming, participants had a larger P2 amplitude than under stranger priming after viewing the painful pictures (kin priming: 4.497 ± 0.266; stranger priming: 4.076 ± 0.254; *P* = .015), whereas no such significant difference was seen for the nonpainful pictures (kin priming: 4.404 ± 0.273; stranger priming: 4.473 ± 0.269; *P* = .710).

#### EEG time-frequency results

**Figure 7.**
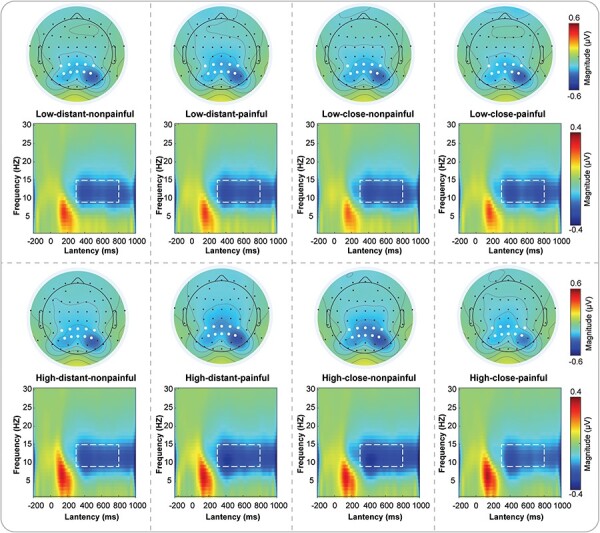
Time-frequency responses during the memory judgment phase.

Supplementary Table S3 provides descriptive statistics (*M* ± SE) of the α-ERD magnitudes in response to memory judgment stimuli under all conditions. Supplementary Table S4 contains a complete list of all statistical comparisons. Grand average TFDs during the memory judgment phase are shown in Fig. 7.

TFDs of neural responses were examined for distant distance nonpainful picture, distant distance painful picture, close distance nonpainful picture, and close distance painful picture conditions in response to low-load letter strings and high-load letter strings during the memory judgment phase. The color scale represents the relative increase or decrease in oscillatory magnitude compared to a prestimulus interval (−200 to 0 ms). The signals were measured at parietal electrodes (CP3, CP1, CPz, CP2, CP4, P3, P1, Pz, P2, and P4). Both high- and low-load stimuli elicited a sustained α-ERD response characterized by oscillations in the alpha frequency range (9–15 Hz) and a latency window of 300–800 ms, indicated by dashed line rectangles. The electrodes used to evaluate the α-ERD magnitude were marked with enlarged dots on the scalp topographies.

For α-ERD, a three-way repeated measures ANOVA revealed a significant two-way interaction effect between distance and painfulness [*F*(1115) =6.90, *P* = .010, *η*_p_^2^ = 0.057] and a three-way interaction effect among load, distance, and painfulness [*F*(1115) = 5.56, *P* = .020, *η*_p_^2^ = 0.046]. We conducted three two-way repeated measures ANOVAs with two factors by fixing another factor, to understand the three-way interaction. We discovered a significant two-way interaction, namely, for high load, there was a significant interaction between distance and painfulness [*F*(1115) = 10.52, *P* = .002, *η*_p_^2^ = 0.084]. When we analyzed the interaction between distance and painfulness further, the results revealed that for high-load conditions, under kin priming, participants had greater α-ERD magnitudes after viewing the nonpainful pictures than after viewing painful pictures (painful pictures: −0.207 ± 0.033; nonpainful pictures: −0.271 ± 0.046; *P* = .007), whereas no such significant difference in α-ERD magnitudes was seen for the stranger priming (painful pictures: −0.257 ± 0.041; nonpainful pictures: −0.229 ± 0.037; *P* = .268). At the same time, for high load, under stranger priming, participants had lower α-ERD magnitudes than under kin priming when viewing the nonpainful pictures (kin priming: −0.271 ± 0.046; stranger priming: −0.229 ± 0.037; *P* = .031), and under the stranger priming, participants had greater α-ERD magnitudes than under kin priming after viewing the painful pictures (kin priming: −0.207 ± 0.033; stranger priming: −0.257 ± 0.041; *P *= .008).

## Discussion

### EfP phase

We investigated whether and how WM load and SD interacted to modulate the behavioral and brain response associated with EfP using EEG. We analyzed the effects on subjective ratings and electrophysiological responses of healthy participants after viewing pictures depicting the pain and nonpain of others at varying SDs. To the best of our knowledge, the results of this study are the first to demonstrate the interactive influence of WM load and SD on the processing of others’ pain. Specifically, we found that high WM load and greater SD primarily facilitate the early processing of others’ pain, as indicated by the N2, and θ-ERS components. On the other hand, low WM load and close SD promoted late-stage processing of others’ pain, as reflected in the ratings of pain intensity, unpleasantness, P3, and LPP components. Several key findings emerged during the EfP phase: first, the N2 results indicate that compared to low WM load, high WM load enhanced emotional sharing of strangers’ pain although it did not affect emotional sharing of kin’s pain, regardless of individuals’ cognitive resource allocation. Furthermore, under conditions of high WM load, individuals showed greater sensitivity to emotional sharing (demonstrated by N2) of strangers’ pain compared to kin’s pain, whereas SD did not impact the participants’ emotional sharing of others’ pain under low WM load. Second, the findings of θ-ERS reveal that relative to low WM load, high WM load promoted emotional sharing of pain when empathizing with kin, whereas emotional sharing of pain in strangers was not modulated by cognitive resource allocation. Lastly, individuals rated the intensity of pain higher when empathizing with kin than with strangers. Under high WM load, individuals also rated unpleasantness higher when empathizing with kin than with strangers. Additionally, close SD resulted in larger P3 and LPP components. Furthermore, low WM load also induced larger LPP components. These results indicate that low WM load and close SD may facilitate late-stage cognitive processing and evaluation of others’ pain.

Previous ERP research proposed a temporal division of EfP into two stages: an early stage of automatic processing involved in sharing others’ emotion and a later stage of cognitive processing and evaluation (Han et al. 2008). Previous research has posited that N2 components are associated with sharing others’ emotions about EfP (Groen et al. 2013). Based on these views and our research findings, we think that when processing stranger’s pain, our participants exhibited significantly higher N2 amplitudes under high WM load than low load. According to the load theory (Lavie et al. 2004, Ahmed et al. 2012), under high cognitive load, individuals are more inclined to process distracting stimuli, such as painful stimuli, which enables them to better process others’ emotions and elicit stronger N2 responses. However, when processing kin’s pain, the level of WM/cognitive load does not affect an individual’s perception of somatosensory pain. This suggests that sharing emotions of kin’s pain is not influenced by the allocation of cognitive resources because the closer SD allows participants to empathize with their kin’s pain regardless of the cognitive load. Thus, the emotional sharing of kin’s pain is experienced similarly well regardless of the WM load. Furthermore, under high WM load, the N2 amplitude generated when processing stranger’s pain was significantly greater than that for processing kin’s pain. However, under low WM load, there was no significant difference in the N2 amplitude between processing kin’s pain and stranger’s pain. This indicates that under high cognitive load, participants were more capable of sharing emotions with strangers’ pain but exhibited weaker emotional sharing for kin’s pain. Conversely, when cognitive resources were relatively ample, sharing emotions for the pain of both kin and strangers was unaffected. This suggests that the allocation of cognitive resources alters individuals’ emotional sharing for EfP. We postulate that based on the threat value of pain hypothesis, the pain of others may represent an impending threat and trigger a defensive response, so others’ pain can serve as a threatening signal to escape from or avoid (Yamada and Decety 2009, Decety et al. 2010, 2012). Consequently, perceiving/sharing another’s emotions may trigger vicarious anxiety about threats and danger, thereby aiding in self-preservation. Because humans are highly concerned about our kin’s pain, when cognitive resources are limited, individuals may allocate more resources to alleviating their kin’s pain rather than immersing in sharing emotions. Thus, under conditions of limited cognitive resources, compared to emotional sharing for strangers’ pain, individuals exhibit weaker emotional sharing for their kin’s pain to conserve resources for helping alleviate their kin’s distress. The results, however, indicate that under low cognitive load, when cognitive resources are relatively abundant, participants can share emotions for both pain of kin and strangers equally. According to the threat value of pain hypothesis (Coll et al. 2012), during this period, participants have more resources to cope with the threats posed by both the pain of kin and strangers, allowing equal EfP for others’ pain regardless of SD.

Based on the differences in N2 amplitude, we speculate that higher WM load leads to weaker emotional sharing when individuals face the pain of kin compared to strangers. This results in greater P3 and LPP amplitudes during the later stages of EfP when individuals empathize with the pain of kin. The results for P3 and LPP are consistent with both pain intensity and pain unpleasantness. A substantial body of EEG research has consistently demonstrated the impact of others’ pain on the subsequent P3 and LPP components, indicating that others’ pain effectively modulates the later parietal components (Coll 2018; Li et al. 2020b, Polich 2007, Schupp et al. 2006). P3 and LPP responses are commonly understood to reflect sustained attentional processing and cognitive evaluation of stimuli that hold motivational significance (Cheng et al. 2012, Coll 2018). In the context of empathy, these processes are believed to contribute to social understanding and emotional regulation, thereby facilitating the empathic response. Furthermore, low WM load induced larger LPP amplitudes. This suggests that lower WM load facilitated the processing of others’ pain in the late stage, which requires more cognitive resources. Under conditions of low WM load, individuals use fewer cognitive resources, allowing for greater evaluation and judgment of others’ pain. From an evolutionary perspective, our priority of attention toward kin far outweighs that toward strangers.

Studies have found a strong correlation between θ-ERS and self-displeasure, and this oscillation contributes to perceiving and sharing of the emotional states of others during EfP (Mu et al. 2008). In our study, when processing kin’s pain, participants exhibited significantly larger θ-ERS magnitudes under high WM load compared to low load. However, when processing stranger’s pain, θ-ERS was not modulated by WM load. Additionally, we found that under high WM load when processing kin’s pain, the participant’s θ-ERS oscillation showed a significant positive correlation with the EC scale scores in the IRI. This means that individuals with higher scores on the EC scale in the IRI had stronger θ-ERS oscillations in response to kin’s pain under high WM load. EC is considered the degree of attention and care individuals have for others’ emotions and needs, and the tendency to EC is associated with experiencing vicarious anxiety (Shu et al. 2017), thereby resulting in more unpleasantness. Thus, we propose that for the sharing of emotional dimensions, a high WM load facilitates an individual’s emotional sharing with their kin’s pain, and the higher their EC is, the more vicarious anxiety they have, resulting in the greater unpleasantness experienced in response to kin’s pain under a high WM load. It is therefore possible that a high WM load causes individuals to feel powerless and have vicarious anxiety toward their kin’s pain, leading to increased unpleasantness. However, the WM load does not affect the sharing of emotional dimensions in response to pain in strangers. This may be due to individuals’ lower concern for strangers, resulting in less vicarious anxiety and unpleasant emotions, which are not influenced by WM load.

### Memory judgment phase

Regarding memory judgment, we investigated whether and how SD and EfP would interact to modulate the behavioral and brain response associated with the performance of WM via the use of EEG. We analyzed the main and interaction effects on healthy participants’ behavioral performance (RT and ACC) and electrophysiological responses to the memory judgment stimuli after they viewed photos depicting others’ pain and nonpain at varying SDs. To the best of our knowledge, our findings are the first to demonstrate the interactive influence of SD and EfP on individuals’ WM performance supported by behavioral and neural data. First, regardless of whether the priming was kin- or stranger-related, participants exhibited longer RT in evaluating memory judgment stimuli after viewing painful photos than after viewing nonpainful photos, indicating that others’ pain weakened the participant’s cognitive performance. Second, under low-load conditions with kin priming, viewing painful pictures resulted in higher ACC during the memory judgment phase than viewing nonpainful pictures, suggesting that under low-load conditions, participants’ kin’s pain enhanced their cognitive performance. However, under high-load conditions, kin’s pain led to lower ACC and smaller α-ERD than kin’s nonpain, indicating that under high-load conditions, kin’s pain weakened participant’s cognitive performance during the memory judgment phase. Lastly, under high-load conditions, compared to stranger’s pain, kin’s pain led to lower ACC, higher P2 amplitude, and smaller α-ERD during the memory judgment phase, indicating that under high-load conditions with others’ pain, closer SD weakened the participant’s cognitive performance.

Regardless of the type of priming, participants exhibited shorter RTs in judging memory stimuli after viewing others’ painful photos. Studies have found that the pain of others impairs WM performance (Chen et al. 2017), and the results of our study confirm this observation in terms of RT. The impairment of WM performance due to the pain of others may be attributed to the activation of pain-related brain regions, similar to the activation of self-pain (Jackson et al. 2005, Bernhardt and Singer 2012, Meng et al. 2013). This activation triggers thoughts of potential danger prompting individuals to regulate or reallocate cognitive resources to cope with the perceived threat (Schoofs et al. 2009, Sanchez 2011, Chen et al. 2017). In our study, cognitive resources were more stretched during others’ pain than during others’ nonpain. Consequently, under pain conditions, individuals required more attention to memory judgment than during nonpain conditions, resulting in longer RT.

The results of ACC during the memory judgment phase indicate that under low-load conditions, kin’s pain leads to higher ACC than kin’s nonpain. The processing efficiency theory postulates that WM system resources are consumed in part by anxiety in individuals, and this anxiety often impairs the performance of “difficult” tasks (especially under test conditions) (Eysenck and Calvo 1992). The amount of WM available for cognitive activities is proportionately diminished when anxiety takes up limited resources. As a result, cognitive functions become less efficient when the available resources are insufficient to meet task demands. However, according to the inverted-U theory of acute stress, moderate levels of stress can enhance an individual’s effort and performance outcomes in tasks of lower difficulty that demand fewer cognitive resources. Moreover, there exists a substantial convergence between stress and anxiety, in terms of both their emotional components and the underlying neurocircuitry. Therefore, we propose that in tasks that require less cognition, the empathy for kin’s pain may to some extent result in more vicarious anxiety compared to kin’s nonpain conditions. In this way, moderately elevated levels of anxiety may enhance individual efforts resulting in better cognitive performance during memory judgment episodes. However, when tasks with high WM load require more cognitive resources compared to the kin’s nonpain condition, kin’s pain leads to lower ACC. This suggests that while kin’s pain results in more vicarious anxiety than kin’s nonpain, it also hinders or interrupts a participant’s cognitive resources during this phase. Thus, lower ACC is the result of not having enough cognitive resources to process the target stimuli during the memory judgment phase, where greater cognitive resources are needed. This point is further supported by the results of α-ERD, where under high-load conditions, the α-ERD generated by the nonpain condition is greater than that generated by the kin’s pain. Based on the inhibition hypothesis, it is posited that α-ERS signifies the inhibition of irrelevant or interfering cognitive processes, whereas α-ERD denotes a release from inhibition. Therefore, a greater α-ERD predicts better performance in memory judgment because it is linked to improved cognitive processing in the task-relevant brain areas (Haegens et al. 2011, Klimesch 2012). In our research, under the kin’s nonpain condition, the task-relevant neural responses (α-ERD) of the participants exhibited heightened activation to such an extent that their ACC in discriminating the WM target stimuli was significantly improved. However, when viewing kin’s pain, more vicarious anxiety was induced, which diminished activation in task-relevant brain responses contributing to a decline in the participant’s ability to discriminate WM target stimuli, resulting in reduced ACC.

Under tasks with high WM load, kin’s pain led to lower ACC during the memory judgment phase than under stranger pain conditions, which suggests that empathizing with kin’s pain induces more vicarious anxiety than during the stranger pain condition to some extent. This effect resulted in a greater need for attentional resources during the memory judgment phase and thus lowered ACC. The results of P2 and α-ERD also support this possibility at the neural level. Specifically, when experiencing high loads, under kin priming, participants exhibited larger P2 amplitudes than during stranger priming after viewing painful photos. Previous research has suggested that in the context of WM tasks, a larger P2 component may reflect increased attentional demands (Gevins et al. 1996, Lijffijt et al. 2009, Lubitz et al. 2017). Under high loads, when participants watched and imagined their kin’s pain, more cognitive resources may have been consumed, leading to a greater need for attentional resources during the memory judgment phase, as reflected by the amplitude of the P2 component. Furthermore, during high loads, under the kin priming, participants had smaller magnitudes of α-ERD than during stranger priming after viewing painful photos. When experiencing high loads while empathizing with the kin’s pain, the α-ERD during the memory judgment phase was smaller than when viewing a stranger’s pain. According to the inhibition hypothesis, this may indicate that during high WM load tasks that require significant cognitive resources, participants are more prone to empathizing with the negative emotions associated with kin’s pain than with a stranger’s pain. Thus, the participants may have had more difficulty in disengaging from these negative emotions, leading to the emergence of relatively stronger vicarious anxiety. Consequently, this more substantial anxiety may have consumed more cognitive resources, thereby interfering with and impeding the activation of task-related brain regions. As a result, the smaller α-ERD during the memory judgment phase ensued, ultimately leading to lower task ACC.

Consistent with previous research findings, larger N3 amplitudes were observed under high-load conditions. Extensive research on the N3 component has identified it as an indicator of “object recognition.” Specifically, the more challenging the identification of the target, the greater the elicited amplitude of the N3 component (Maguire et al. 2013). In our study, the identification difficulty of target WM stimuli was higher in high-load WM tasks than in low-load ones, resulting in a larger N3 component.

## Limitations and future directions

Although the findings of this study demonstrate the mutual influence of WM and EfP at both behavioral and neural levels during the same temporal process, enhancing our understanding of how humans dynamically adapt to the complexities of social life, the study is not without limitations. First, our sample was relatively homogeneous, consisting solely of college students aged 18–28 years. This group, being generally free from significant life stressors and possessing robust physiological and psychological resources, is naturally more predisposed to experiencing empathy and cognitive processing. Thus, the generalizability of our findings to broader populations remains uncertain. Future research should include individuals from various age groups, particularly children and older adults, to examine how emotional and cognitive interactions influence one another across different life stages. Second, in our study, EfP was primarily induced by having participants view static images and engage in imaginative processes. However, in everyday life, EfP often arises from dynamic, multifactorial interactions. To enhance ecological validity, future studies should employ wearable neurophysiological recording devices to capture real-time cognitive and empathic interactions under varying cognitive loads in more naturalistic settings. Third, while our behavioral and electrophysiological studies elucidated the mutual influence of WM and EfP during the same temporal process, with SD emerging as a crucial moderating factor, the low spatial resolution of EEG methods limits our ability to thoroughly explore the underlying neural mechanisms and circuits. Future research should integrate EEG with functional Magnetic Resonance Imaging to investigate these mutual influences more comprehensively from psychological, cognitive, temporal, and spatial perspectives. We anticipate that future research building on these studies will explore how affective and cognitive interactions occur in real-life scenarios where individuals’ cognitive resources are engaged to varying degrees and how SD specifically affects these interactions. Extending this line of inquiry can promote social harmony. For instance, understanding how doctors empathize with individuals at different SDs in high-load work environments and how different states of empathy affect their job performance could inform the development of more effective healthcare management policies to mitigate conflicts between healthcare providers and patients.

## Conclusion

Our research findings suggest that within the same temporal process, the influence of WM and EfP is bidirectional, and this bidirectionality is further influenced by the level of SD. Specifically, the interaction between WM and SD affects individuals’ processing of others’ pain. WM with high load and distant SD facilitates early processing of others’ pain, whereas WM with low load and close SD promotes late processing of others’ pain. Furthermore, the interaction between others’ pain and SD during the same processing phase affects performance in ongoing WM tasks. Specifically, kin’s pain (compared to kin’s nonpain) improves individuals’ performance in WM with low load, but it diminishes their performance in WM with high load. However, regardless of the load level of the WM tasks, a stranger’s pain or nonpain did not significantly affect individual performance in the tasks. Moreover, kin’s pain weakens individuals’ performance in WM with high load more than stranger’s pain, whereas the level of SD did not significantly affect the participants’ performance in WM with low load.

## Supplementary Material

nsae061_Supp

## Data Availability

The study’s data and code are publicly available at https://osf.io/86gyx/ on the Open Science Framework.
