# Supplementary material for: The mutual influences between working memory and empathy for pain: the role of social distance
Source: Soc Cogn Affect Neurosci. 2024 Sep 6;19(1):nsae061. doi: 10.1093/scan/nsae061 (PMC11451274; doi:10.1093/scan/nsae061)
Supplement: nsae061_Supp [file nsae061_supp.zip › scan-24-031-File009.docx]

***GPower_Output***

*The detailed output parameters are as follows:*

- *Noncentrality parameter λ: 16.000*
- *Critical F: 2.098*
- *Numerator df: 7.000*
- *Denominator df: 105*
- *Total sample size: 16*
- *Actual power: 0.820*

***Supplementary Table 1***

*Subjective ratings (pain intensity and unpleasantness), time-domain responses (maximal amplitudes of N2, P3, and mean amplitudes of LPP) and time frequency responses (θ-ERS oscillation) evoked by painful and non-painful pictures in empathy for pain phase.*

|  | **Low load (M ± *SE*)** | | | | **High load (M ± *SE*)** | | | |
| --- | --- | --- | --- | --- | --- | --- | --- | --- |
|  | **Distant** | | **Close** | | **Distant** | | **Close** | |
|  | **Painful pictures** | **Nonpainful pictures** | **Painful pictures** | **Nonpainful pictures** | **Painful pictures** | **Nonpainful pictures** | **Painful pictures** | **Nonpainful pictures** |
| **Pain Intensity rating** | 5.95±0.09 | 1.77±0.06 | 6.13±0.09 | 1.81±0.06 | 5.95±0.09 | 1.8±0.06 | 6.1±0.09 | 1.84±0.06 |
| **Unpleasantness rating** | 5.52±0.14 | 0.82±0.07 | 5.73±0.15 | 0.85±0.07 | 5.54±0.15 | 0.86±0.07 | 5.67±0.15 | 0.86±0.07 |
| **N2 amplitude (μV)** | -10.39±0.41 | -11.01±0.43 | -10.17±0.42 | -10±0.42 | -10.88±0.44 | -10.76±0.45 | -10.25±0.42 | -10.25±0.40 |
| **P3 amplitude (μV)** | 7.82±0.42 | 6.35±0.42 | 8.32±0.41 | 6.89±0.39 | 7.70±0.43 | 6.31±0.38 | 8.17±0.42 | 6.78±0.41 |
| **LPP amplitude (μV) (uV) (μV)** | 4.02±0.29 | 2.74±0.27 | 4.53±0.28 | 3.5±0.25 | 3.57±0.28 | 2.66±0.25 | 4.43±0.28 | 3.39±0.26 |
| **θ-ERS oscillation (μV)** | 0.35±0.02 | 0.29±0.02 | 0.31±0.03 | 0.30±0.02 | 0.34±0.02 | 0.29±0.2 | 0.36±0.03 | 0.28±0.02 |

***Supplementary Table 2***

*Summary of statistical analysis of behavioral, time-domain responses and time frequency data in empathy for pain phase. Note: The significant (p < 0.05) comparisons were shown in boldface. df:(1,115)*

|  | **Pain Intensity rating** | | | **Unpleasantness rating** | | | **N2** | | | **P3** | | | **LPP** | | | **θ-ERS** | | | |
| --- | --- | --- | --- | --- | --- | --- | --- | --- | --- | --- | --- | --- | --- | --- | --- | --- | --- | --- | --- |
|  | ***F*** | ***P*** | ***η_p_^2^*** | ***F*** | ***P*** | ***η_p_^2^*** | ***F*** | ***P*** | ***η_p_^2^*** | ***F*** | ***P*** | ***η_p_^2^*** | ***F*** | ***P*** | ***η_p_^2^*** | ***F*** | ***P*** | ***η_p_^2^*** |  |
| **Load** | 0.02 | 0.904 | 0.000 | 0.01 | 0.968 | 0.000 | 1.76 | 0.187 | 0.015 | 0.69 | 0.407 | 0.006 | **4.00** | **0.048** | **0.034** | 0.39 | 0.533 | 0.003 |  |
| **Distance** | **32.41** | **<0.001** | **0.220** | **14.20** | **<0.001** | **0.110** | **27.33** | **<0.001** | **0.192** | **19.77** | **<0.001** | **0.147** | **72.80** | **<0.001** | **0.388** | 0.38 | 0.538 | 0.003 |  |
| **Painfulness** | **1789.76** | **<0.001** | **0.940** | **1299.42** | **<0.001** | **0.919** | 0.64 | 0.426 | 0.006 | **147.40** | **<0.001** | **0.562** | **103.43** | **<0.001** | **0.474** | **25.29** | **<0.001** | **0.180** |  |
| **Load × Distance** | 0.90 | 0.346 | 0.008 | **4.21** | **0.042** | **0.030** | 0.07 | 0.799 | 0.001 | 0.09 | 0.769 | 0.001 | 1.86 | 0.175 | 0.016 | 0.41 | 0.524 | 0.004 |  |
| **Load × Painfulness** | 1.04 | 0.310 | 0.009 | 0.60 | 0.442 | 0.005 | 2.55 | 0.113 | 0.022 | 0.09 | 0.767 | 0.001 | 1.37 | 0.244 | 0.012 | 2.20 | 0.141 | 0.019 |  |
| **Distance × Painfulness** | **25.21** | **<0.001** | **0.180** | **14.27** | **<0.001** | **0.110** | 3.07 | 0.083 | 0.026 | 0.00 | 0.952 | 0.001 | 0.23 | 0.636 | 0.002 | 0.44 | 0.511 | 0.004 |  |
| **Load × Distance ×Painfulness** | 0.52 | 0.472 | 0.005 | 0.95 | 0.332 | 0.008 | **8.92** | **0.003** | **0.072** | 0.02 | 0.889 | 0.001 | 2.10 | 0.151 | 0.018 | **5.40** | **0.022** | **0.045** |  |

***Supplementary Table 3***

*Key responses(reaction times (RT) and accuracies (ACC)), time-domain responses (maximal amplitudes of N3and P2) and time frequency responses (α-ERD oscillation) evoked by memory judgment stimuli in memory judgment phase.*

|  | **Distant (M ± *SE*)** | | | | **Close (M ± *SE*)** | | | |
| --- | --- | --- | --- | --- | --- | --- | --- | --- |
|  | **Nonpainful** | | **Painful** | | **Nonpainful** | | **Painful** | |
|  | **High load** | **Low load** | **High load** | **Low load** | **High load** | **Low load** | **High load** | **Low load** |
| **RT (ms)** | 1994.84±64.29 | 1215.59±37.75 | 2125.12±72.71 | 1330.42±35.83 | 1975.04±65.11 | 1271.86±35.56 | 2049.04±69.99 | 1323.05±36.15 |
| **ACC (%)** | 82.5±0.9 | 86.8±0.7 | 81.2±1.1 | 86.9±0.8 | 82.7±0.9 | 79±0.7 | 77±0.9 | 85.7±0.8 |
| **N3 amplitude (μV)** | -4.06±0.26 | -3.63±0.29 | -4.49±0.26 | -3.45±0.29 | -4.19±0.27 | -3.85±0.27 | -4.23±0.28 | -3.85±0.29 |
| **P2 amplitude (μV) (uV) (μV)** | 4.47±0.26 | 4.62±0.28 | 4.08±0.25 | 4.69±0.29 | 4.4±0.27 | 4.61±0.27 | 4.5±0.27 | 4.44±0.27 |
| **α-ERD oscillation (μV) （dB）** | -0.229±0.04 | -0.22±0.04 | -0.257±0.04 | -0.234±0.04 | -0.271±0.05 | -0.232±0.04 | -0.207±0.03 | -0.226±0.04 |

***Supplementary Table 4***

*Summary of statistical analysis of behavioral, time-domain and time frequency data in memory judgment phase.* *Note: The significant (p < 0.05) comparisons were shown in boldface. df:(1,115)*

|  | **RTs** | | | **ACCs** | | | **N3** | | | **P2** | | | α-ERD | | |
| --- | --- | --- | --- | --- | --- | --- | --- | --- | --- | --- | --- | --- | --- | --- | --- |
|  | ***F*** | ***P*** | ***η_p_^2^*** | ***F*** | ***P*** | ***η_p_^2^*** | ***F*** | ***P*** | ***η_p_^2^*** | ***F*** | ***P*** | ***η_p_^2^*** | ***F*** | ***P*** | ***η_p_^2^*** |
| **Load** | **205.23** | **<0.001** | **0.641** | **40.44** | **<0.001** | **0.26** | **9.24** | **0.003** | **0.074** | 3.03 | 0.084 | 0.026 | 0.62 | 0.432 | 0.005 |
| **Distance** | 1.31 | 0.254 | 0.011 | **85.40** | **<0.001** | **0.426** | 1.21 | 0.273 | 0.01 | 0.66 | 0.798 | 0.001 | 0.02 | 0.884 | 0.000 |
| **Painfulness** | **36.16** | **<0.001** | **0.239** | 0.01 | 0.928 | 0.000 | 0.39 | 0.532 | 0.003 | 1.27 | 0.262 | 0.011 | 0.42 | 0.521 | 0.004 |
| **Load × Distance** | **17.50** | **<0.001** | **0.132** | **13.16** | **<0.001** | **0.103** | 2.94 | 0.089 | 0.025 | 3.24 | 0.074 | 0.027 | 0.08 | 0.777 | 0.001 |
| **Load × Painfulness** | 0.67 | 0.414 | 0.006 | **91.60** | **<0.001** | **0.443** | 2.07 | 0.153 | 0.018 | 0.30 | 0.584 | 0.003 | 0.86 | 0.356 | 0.007 |
| **Distance × Painfulness** | **6.69** | **0.011** | **0.055** | 1.80 | 0.183 | 0..015 | 0.28 | 0.595 | 0.002 | 0.50 | 0.481 | 0.004 | **6.90** | **0.01** | **0.057** |
| **Load × Distance ×Painfulness** | 0.03 | 0.856 | 0.000 | **81.76** | **<0.001** | **0.416** | 1.83 | 0.179 | 0.016 | **5.30** | **0.023** | **0.044** | **5.56** | **0.02** | **0.046** |
